# Prognostic Value of Diastolic Dysfunction Derived From D-SPECT in Coronary Artery Disease Patients With Normal Ejection Fraction

**DOI:** 10.3389/fcvm.2021.700027

**Published:** 2021-07-15

**Authors:** Bin Xu, Lu Liu, Fuad A. Abdu, Guoqing Yin, Abdul-Quddus Mohammed, Siling Xu, Xian Lv, Rui Fan, Cailin Feng, Tingting Shi, Wen Zhang, Yawei Xu, Haidong Cai, Fei Yu, Wenliang Che

**Affiliations:** ^1^Department of Cardiology, Shanghai Tenth People's Hospital, Tongji University School of Medicine, Shanghai, China; ^2^Medical College of Soochow University, Soochow University, Suzhou, China; ^3^Department of Nuclear Medicine, Shanghai Tenth People's Hospital, Tongji University School of Medicine, Shanghai, China; ^4^Department of Cardiology, Shanghai Tenth People' s Hospital Chongming Branch, Shanghai, China

**Keywords:** diastolic dysfunction, D-SPECT, peak filling rate, prognosis, CAD

## Abstract

Diastolic dysfunction (DD) with normal systolic function has been elucidated to be associated with heart failure and worse prognosis. The recently introduced single photon emission computed tomography (SPECT) with dedicated cardiac cadmium-zinc-telluride (CZT) cameras (D-SPECT) is a novel method to quantitate left ventricular functional parameters. We aimed to evaluate the prognostic value of DD derived from D-SPECT in coronary artery disease (CAD) patients with normal ejection fraction. All CAD patients who underwent D-SPECT and invasive coronary angiography within 3 months were considered. DD was defined as peak filling rate (PFR) <2.1 end diastolic volume (EDV, ml)/s according to the D-SPECT results. Patients were divided into three groups: group 1 (*n* = 226)—normal PFR; group 2 (*n* = 67)—ischemia-related DD (abnormal stress PFR and normal rest PFR); and group 3 (*n* = 106)—rest DD (abnormal rest PFR). The primary clinical endpoint of the present study was a composite of heart failure events (HFE). A total of 399 consecutive CAD patients with normal systolic function undergoing stress D-SPECT were analyzed. The incidence rates of HFE among the three groups were 4.0, 7.5, and 11.3%, respectively. Cox regression analysis showed that the multivariate predictors of HFE were rest PFR, diabetes mellitus, obesity, and old age. DD derived from D-SPECT in CAD patients with normal ejection fraction is predictive of HFE.

## Introduction

Diastolic dysfunction (DD) with normal systolic function has been elucidated to be associated with development of heart failure (HF) and is predictive of all-cause mortality (1–3). The prevalence of DD with normal systolic function in the general adult population is approximately 20–30% and increases with old age, diabetes mellitus (DM), and the presence of cardiovascular comorbidities, such as hypertension, obesity, and coronary artery disease (CAD) (1, 4, 5). Advanced DD with normal ejection fraction showed evidence of structural remodeling compared with subjects with normal or mild DD (4). In addition, the extent and severity of CAD is associated with measures of worsening diastolic function (6, 7). Therefore, early identification of DD might be of significant value in risk stratification of CAD patients with normal ejection fraction (4, 6, 8, 9).

Echocardiography has so far played a central role in the evaluation of left ventricular (LV) diastolic function and has been recommended in the American Society of Echocardiography (ASE) guideline (10). It has been validated that DD evaluated by echocardiography is associated with poor prognosis (1–3, 6). On the other hand, the recently introduced gated single photon emission computed tomography (SPECT) with dedicated cardiac cadmium-zinc-telluride (CZT) cameras (D-SPECT) demonstrated the feasibility to simultaneously quantitate myocardial perfusion image (MPI), as well as LV functional parameters (11–13). D-SPECT, compared with conventional SPECT, has more favorable sensitivity, specificity, and accuracy with a low-dose and ultra-fast protocol (13–15). In addition, D-SPECT derived measures of LV filling dynamics showed significant correlation with diastolic parameters derived from echocardiography and cardiac magnetic resonance (CMR) (11, 12).

Our aim is to evaluate the prognostic value of DD derived from D-SPECT in CAD patients with normal ejection fraction.

## Methods

### Study Population

We observationally analyzed 399 consecutive patients admitted to Shanghai Tenth People's Hospital from April 2017 to July 2019. Stress D-SPECT MPI was evaluated followed by invasive coronary angiography (CAG) within 3 months. Every patient in the present study was diagnosed as CAD patient with CAG or who had a history of CAD that was defined as previous coronary revascularization and/or healed myocardial infarction. According to the echocardiography, all patients with abnormal left ventricular ejection fraction (LVEF <50%) were excluded. Patients with severe valvular dysfunction, cardiomyopathy, and atrial fibrillation were also excluded. Moreover, patients with severe renal disease or concomitant hepatic and malignant tumor with an expected survival time of <1 year were excluded. We recorded the basic information (e.g., age, gender, heart rate, and blood pressure), medical history (e.g., DM and hypertension), and the medication data of all the patients. Written informed consent was obtained from every patient. This study protocol was approved by the ethical review board (Shanghai Tenth People's Hospital, Tongji University, Shanghai, China), and all study procedures conformed with the Declaration of Helsinki.

In the present study, DD was defined as peak filling rate (PFR) <2.1 end diastolic volume (EDV, ml)/s according to the D-SPECT results in the absence of an accepted cut-off value of PFR (11). Both rest and stress PFRs were collected in all patients. The study population was categorized into three groups. Group 1 had normal rest and stress PFRs, which are considered completely normal diastolic function. Group 2 enrolled patients with abnormal stress PFR and normal rest PFR, which were deemed to have ischemia-related DD. Group 3 enrolled all patients with abnormal rest PFR.

### Coronary Angiography

CAG was operated based on the standard method, and at least two right coronary artery projections and four left coronary artery projections were performed (16). The coronary angiograms were assessed by at least two experienced cardiologists, and all disagreements were resolved by consensus. Obstructive CAD was defined as stenosis ≥50% in at least one major epicardial vessel.

### D-SPECT Images Acquisition

D-SPECT image acquisition was performed as previously described (17). All patients underwent a single-day rest–stress ^99m^Tc-sestamibi (^99m^Tc-MIBI; HTA Co., Ltd., Beijing, China) protocol performed with a D-SPECT cardiac camera (Spectrum Dynamics, Biosensors, Caesarea, Israel). Our D-SPECT camera consisted of nine rotating pixelated detector columns of CZT crystals. Every detector was furnished with a wide-angle square-hole tungsten collimator and rotated around its central axis during the scanning process focusing on the region of interest (ROI). The spatial and temporal resolutions of the CZT camera are 5.0 mm and 1 ms, respectively, and 8-frame gated acquisitions were used in the present study.

All patients were requested to avoid caffeine, nitrates, calcium channel blockers (CCB), and beta-blockers for 24–48 h prior to testing. All patients underwent pharmacological stress testing by intravenous administration of adenosine triphosphate (ATP, 140 μg/kg·min, for 6 min; Shanghai Shyndec Pharmaceutical Co., Ltd., Shanghai, China). In our nuclear medicine center, pharmacological stress testing was a common practice attributed to logistical reasons. After injection of ^99m^Tc-MIBI for 30 min, patients were instructed to drink three cups of water and consume at least half a chocolate bar to decrease the uptake of subdiaphragmatic activity and enhance the image quality of the inferior wall. ^99m^Tc-MIBI was bolus injected as a perfusion radiotracer by an automatic injector to control the reproducibility and quality of radiotracer infusion (18). Heart rate, blood pressure, 12-lead electrocardiogram, and clinical symptoms were recorded every minute before, during, and after ATP injection. No major side effects occurred during the stress test in all patients.

After the injection of a low dose of radiotracer, pre-scanning was performed to position the heart in the field of view. The rest image acquisition was performed approximately 1 h after ^99m^Tc-MIBI injection (3 MBq/kg) with the patient being seated in the supine position for 6 min. With the intravenous infusion of ATP (140 μg/kg·min, for 6 min), the stress image acquisition was initiated 30 min after the recording of rest image acquisition, and the ^99m^Tc-MIBI (9 MBq/kg) was injected 3 min after ATP. After a 30-minute interval, the stress image acquisition was performed for 6 min. We applied the algorithm “Ordered Subset Expectation Maximization” (OSEM) for image reconstruction, combining the normalize filter of 4 iterations (I) and 32 subsets (S) in D-SPECT scanner for the energy window of 140 keV±10%. There was no scatter correction or attenuation correction applied. With QPS software (Cedars-Sinai Medical Center, LA, CA, USA), images were reoriented into short-axis, horizontal, and vertical long-axis slices.

### Analysis of Perfusion Images

All MPI images were semi-quantitatively interpreted with a 17-segment model, the semi-quantitative analysis was performed by two experienced nuclear cardiologists, and all disagreements were resolved by consensus (Supplementary Figures 1, 2). Each segment was scored based on a five-point scale (0 = normal; 1 = equivocal; 2 = moderate; 3 = severe reduction in radioisotope uptake; 4 = absence of detectable tracer uptake) (19). The summed rest score (SRS) and the summed stress score (SSS) were calculated by adding all 17 segments scores, respectively, in the stress and rest images. The summed difference score (SDS) was obtained by the difference between SSS and SRS. Myocardial ischemia was considered to exist in individual coronary territories while the SSS was ≥4, and the SDS was ≥2 (20, 21).

LV volume and LVEF were automatically calculated using QPS software. Manual editing was performed in patients with inadequate border detection. The LV-filling rate/time curve was computed from the first derivative of the volume/time curve. PFR was defined as the maximum dV/dt value divided by end-diastolic volume per second, and the unit for PFR was EDV/s (18). The time to peak filling (TTPF), expressed in milliseconds, was defined as the interval between end systole and PFR, and the unit for TTPF was milliseconds. MFR/3 represents the mean rate of filling over the first third of diastole.

### Follow-Up

Follow-up data were collected for at least 2 years in the present study after the D-SPECT data collection was commenced. Patients were requested to record their status and contact us as soon as they developed any symptoms after the D-SPECT test. Follow-up was performed by trained cardiologists in Shanghai Tenth People's Hospital. Our patient population was followed up *via* telephone calls, clinical visit, and medical records. If we failed to get connection with the patient, follow-up information would be collected through their treating doctors or family members.

The primary clinical endpoint of the present study was a composite of heart failure events (HFE), including hospital admission with a diagnosis of heart failure, worsening New York Heart Association (NYHA) class, a reduction in LVEF >10%, and cardiovascular death. Heart failure was diagnosed according to the European Society of Cardiology (ESC) guideline characterized by typical symptoms (e.g., breathlessness, ankle swelling, and fatigue) and signs (e.g., elevated jugular venous pressure, pulmonary crackles, and peripheral edema) (22). At the time of follow-up, the treating cardiologists would assess the patient whether NYHA class had deteriorated. Cardiovascular death was defined as death attributed to acute coronary syndrome, severe cardiac arrhythmia, or refractory congestive heart failure.

### Statistical Analysis

Statistical Package for Social Sciences (SPSS) v.22 software (IBM Corp., Armonk, NY, USA) was applied to perform statistical analyses. An independent sample *t*-test was used for intergroup comparisons of numerical variables. Numerical variables were presented as mean±SE with a normal distribution. Categorical variables were expressed as percentages, and the chi-square test and Fisher's exact tests were used for comparisons. To quantify the relative risk of outcomes between three groups, Cox regression models were performed to derive adjusted hazard ratio (HR) for HFE. Covariates in the models were gender, age, obesity, hypertension, DM, hyperlipidemia, LVEF, rest PFR, stress PFR, and ischemia-related DD. All factors univariate analysis with *p* < 0.10 were considered as covariates for multivariable model. For evaluation of HFE, Kaplan–Meier survival analysis was performed, and differences were assessed by the log-rank test. All tests were performed two-sided and identified statistically significant at a *p*-value of <0.05.

## Results

### Baseline Clinical Characteristics

**Figure 1:**
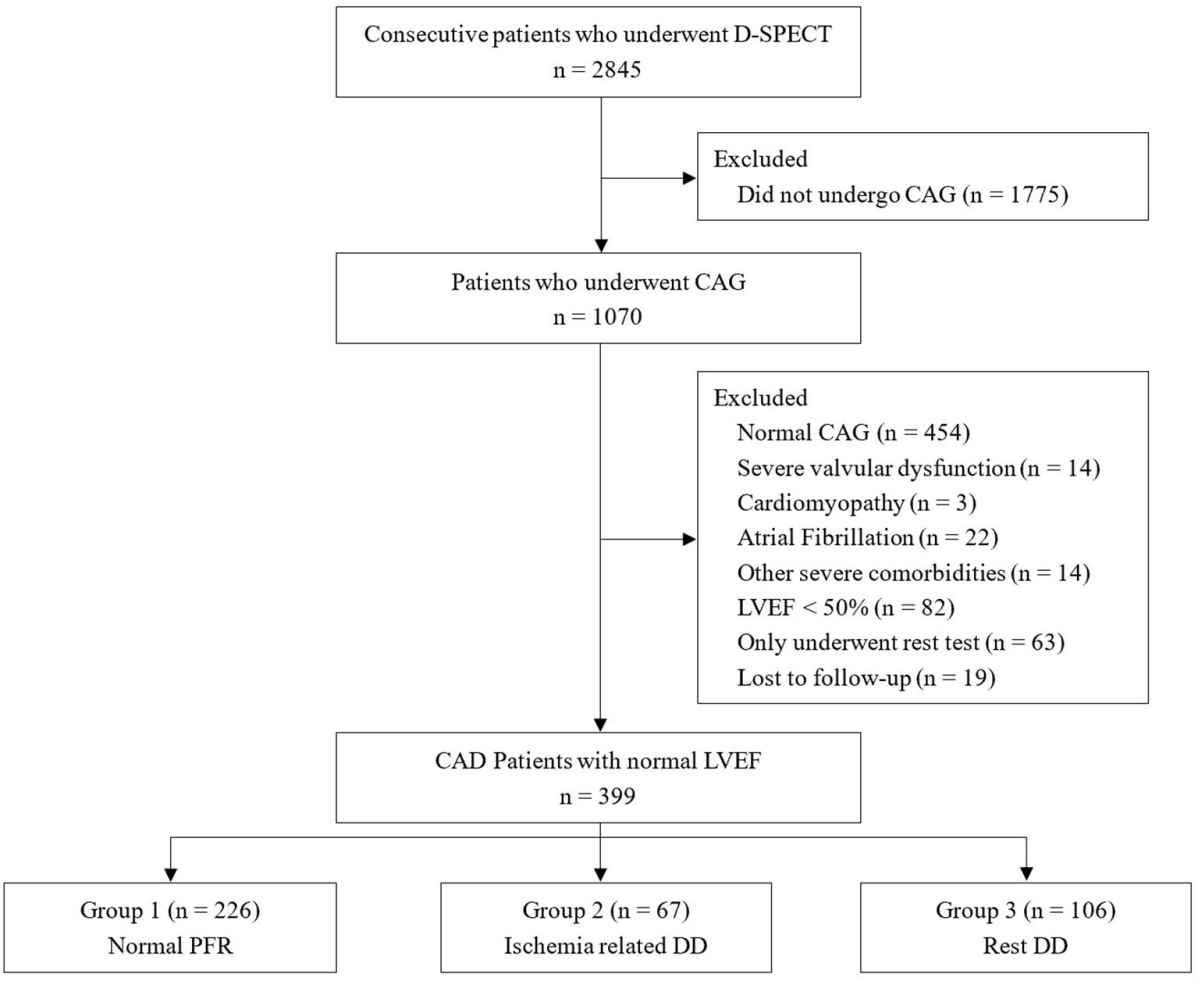
Study flowchart. CAG, coronary angiography; LVEF, left ventricular ejection fraction; CAD, coronary artery disease; DD, diastolic dysfunction.

A total of 399 consecutive CAD patients with normal systolic function undergoing stress D-SPECT were analyzed (Figure 1). Mean age was 64.3 ± 9.3 years. Two hundred twenty-six (56.6%) patients (Group 1) had both normal rest and stress PFRs, 67 (16.8%) patients (Group 2) had normal rest and stress PFRs, and 106 (26.6%) patients (Group 3) had rest DD. Group 1 was more likely to be female and had higher heart rates at admission. Echocardiography data showed that LVEF was higher in group 1 than in group 2 and group 3. There were no statistical differences between three groups in the other baseline characteristics. The baseline characteristics of the patients are listed in Table 1.

**Table 1:** Baseline characteristics of the study population.

**Variables**	**Group 1 (*n* = 226)**	**Group 2 (*n* = 67)**	**Group 3 (*n* = 106)**	***p*-Value**
Female, *n* (%)	87 (38.5)	14 (20.9)	20 (18.9)	< 0.001
Age (years)	64.23 ± 8.85	63.91 ± 10.28	64.76 ± 9.61	0.842
BMI (kg/m^2^)	24.98 ± 3.19	25.15 ± 2.95	24.79 ± 2.52	0.727
DM, *n* (%)	79 (35.0)	17 (25.4)	28 (26.4)	0.16
HLP, *n* (%)	63 (27.9)	19 (28.4)	36 (34.0)	0.512
HTN, *n* (%)	153 (67.7)	47 (70.1)	74 (69.8)	0.917
Smoking history, *n* (%)	52 (23.0)	18 (26.9)	35 (33.0)	0.161
PCI history, *n* (%)	162 (72.0)	48 (71.6)	79 (74.5)	0.874
SBP (mmHg)	134.00 ± 20.02	137.02 ± 21.73	133.24 ± 22.91	0.492
DBP (mmHg)	77.23 ± 12.69	79.28 ± 12.08	76.44 ± 13.42	0.353
HR (bpm)	77.30 ± 11.50	76.66 ± 10.99	73.305 ± 11.25	0.011
TC (mmol/L)	3.78 ± 1.06	3.71 ± 0.94	3.69 ± 0.98	0.756
TG (mmol/L)	1.72 ± 1.41	1.66 ± 1.07	1.73 ± 1.05	0.938
HDL-C (mmol/L)	1.10 ± 0.27	1.09 ± 0.26	1.04 ± 0.25	0.241
LDL-C (mmol/L)	2.08 ± 0.93	1.99 ± 0.89	2.01 ± 0.85	0.697
HBA1c (%)	6.57 ± 1.30	6.33 ± 1.26	6.32 ± 0.90	0.161
cTnT (ng/ml)	0.095 ± 0.512	0.194 ± 0.725	0.195 ± 1.046	0.404
CK-MB (ng/ml)	4.84 ± 19.22	6.87 ± 26.54	5.64 ± 29.75	0.821
MYO (ng/ml)	39.74 ± 59.73	39.02 ± 33.47	41.89 ± 58.85	0.933
NT-proBNP (pg/ml)	218.5 ± 702.0	212.4 ± 369.1	349.3 ± 1330.0	0.419
LVEF (%)	63.3 ± 3.9	61.4 ± 4.9	59.8 ± 6.6	< 0.001

*BMI, body mass index; DM, diabetes mellitus; HLP, hyperlipidemia; HTN, hypertension; PCI, percutaneous coronary intervention; SBP, systolic blood pressure; DBP, diastolic blood pressure; HR, heart rate; TC, total cholesterol; TG, triglyceride; HDL-C, high-density lipoprotein cholesterol; LDL-C, low-density lipoprotein cholesterol; HBA1c, glycated hemoglobin; cTnT, cardiac troponin T; CK-MB, creatine kinase MB; MYO, myoglobin; NT-proBNP, N-terminal pro-B-type natriuretic peptide; LVEF, left ventricular ejection fraction*.

### D-SPECT Parameters

Table 2 shows MPI and LV functional parameters collected by D-SPECT. Group 2 and group 3 had higher EDV and end systolic volume (ESV) in both rest and stress tests. Group 1 had higher LVEF derived from D-SPECT in both rest and stress tests. Rest PFRs of three groups were 2.82 ± 0.76, 2.52 ± 0.98, and 1.71 ± 0.33 EDV/s, whereas stress PFRs of three groups were 2.70 ± 0.58, 1.78 ± 0.27, and 1.92 ± 0.43 EDV/s, respectively. Rest MFR/3 of three groups were 1.25 ± 0.36, 1.14 ± 0.33, and 1.02 ± 0.27 EDV/s, and stress MFR/3 of three groups were 1.27 ± 0.35, 1.06 ± 0.25, and 1.10 ± 0.31 EDV/s. However, LV diastolic function parameter and TTPF showed no statistical differences between three groups in both rest and stress tests. Furthermore, there were no significant differences in ischemic-related indicators including SRS, total perfusion defects (TPD), and transient ischemic dilation (TID) between three groups.

**Table 2:** D-SPECT data of the study population.

**Variables**	**Group 1 (*n* = 226)**	**Group 2 (*n* = 67)**	**Group 3 (*n* = 106)**	***p*-Value**
SRS (median ± IQR)	1.40 ± 3.09	1.46 ± 2.66	2.04 ± 4.42	0.274
SSS (median ± IQR)	3.19 ± 4.71	3.14 ± 4.18	4.03 ± 5.57	0.307
SDS (median ± IQR)	1.80 ± 2.30	1.76 ± 2.14	1.99 ± 2.29	0.729
TPD (%)	4.38 ± 6.38	4.25 ± 5.72	5.22 ± 7.09	0.495
TID	1.09 ± 0.15	1.11 ± 0.12	1.10 ± 0.12	0.677
Rest EDV (ml)	62.43 ± 18.46	75.36 ± 18.54	86.82 ± 27.47	< 0.001
Rest ESV (ml)	20.89 ± 10.11	28.99 ± 11.72	38.49 ± 18.51	< 0.001
Rest LVEF (%)	67.53 ± 12.30	62.55 ± 9.46	56.69 ± 13.21	< 0.001
Rest PER (–EDV/s)	3.71 ± 0.71	3.17 ± 0.57	2.78 ± 0.73	< 0.001
Rest PFR (EDV/s)	2.82 ± 0.76	2.52 ± 0.98	1.71 ± 0.32	< 0.001
Rest TTPF (ms)	178.9 ± 59.0	193.9 ± 69.5	186.8 ± 56.0	0.161
Rest MFR/3 (EDV/s)	1.25 ± 0.36	1.14 ± 0.33	1.02 ± 0.27	< 0.001
Stress EDV (ml)	69.09 ± 20.00	84.72 ± 19.53	93.23 ± 28.37	< 0.001
Stress ESV (ml)	23.94 ± 11.42	35.66 ± 12.38	41.6 ± 20.48	< 0.001
Stress LVEF (%)	66.89 ± 8.14	58.70 ± 9.60	57.45 ± 10.83	< 0.001
Stress PER (–EDV/s)	3.49 ± 0.65	2.88 ± 0.54	2.89 ± 0.69	< 0.001
Stress PFR (EDV/s)	2.70 ± 0.58	1.78 ± 0.27	1.92 ± 0.43	< 0.001
Stress TTPF (ms)	176.4 ± 59.5	191.0 ± 44.0	181.9 ± 47.2	0.144
Stress MFR/3 (EDV/s)	1.27 ± 0.35	1.06 ± 0.25	1.1 ± 0.31	< 0.001

*SRS, summed rest score; SSS, summed stress score; SDS, summed difference score; TPD, total perfusion defects; TID, transient ischemic dilation; EDV, end diastolic volume; ESV, end systolic volume; LVEF, left ventricular ejection fraction; PER, peak ejection rate; PFR, peak filling rate; TTPF, time to peak filling; MFR/3, mean filling rate over first third of diastole*.

### Follow-Up

**Figure 2:**
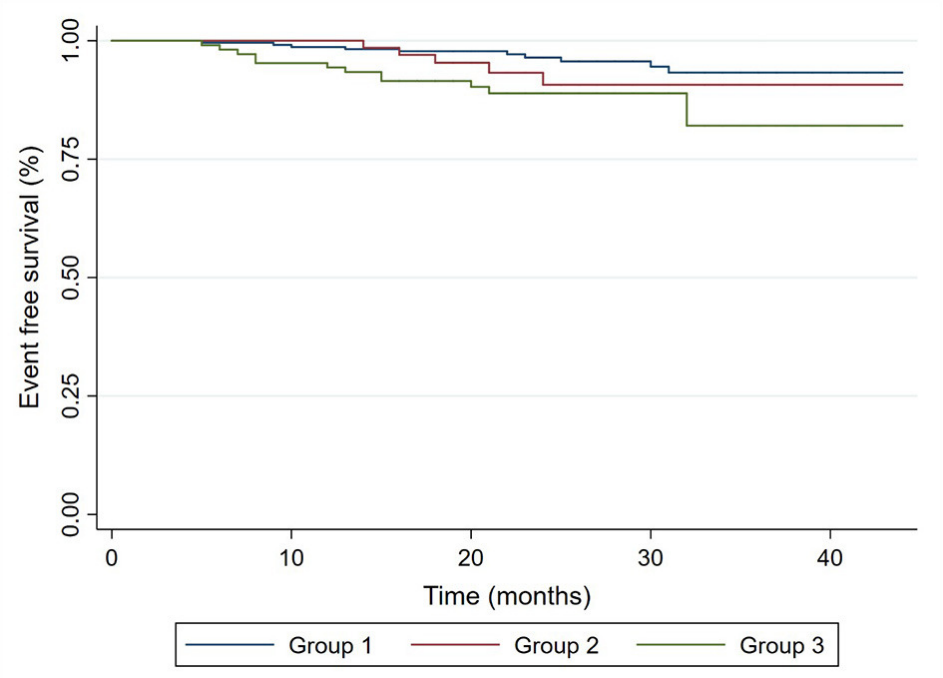
Kaplan–Meier curves for freedom from HFE in CAD patients with normal ejection fraction. Group 1, normal PFR; Group 2, ischemia-related DD; Group 3, rest DD.

Follow-up data were available for 399 patients (95.5%), and 19 patients (4.5%) were lost to follow-up. In the total cohort, 26 (6.5%) HFE were observed, including 26 HF admission, 6 worsening NYHA class, 7 reduction in LVEF >10%, and 1 CV death. The incidence of HFE of group 1 was the lowest (4.0%), followed by group 2 (7.5%), and group 3 had the highest incidence of HFE (11.3%) (*p* = 0.039). Follow-up data are presented in Table 3, and Kaplan–Meier curves for freedom from HFE are shown in Figure 2.

**Table 3:** Heart failure events by group.

**Group**	**HFE**	**Event**	**HF**	**↑NYHA**	**↓LVEF**	**CV**
		**rate (%)**	**admission**	**class**		**death**
1	9	4.00	4	3	1	1
2	5	7.50	2	1	2	0
3	12	11.30	6	2	4	0
Total	26	6.50	12	6	7	1

*HFE, heart failure events; CV death, cardiovascular death*.

### Predictive Factors

The results of Cox regression analysis are displayed in Table 4. Independent predictors of HFE in univariate analysis were rest PFR (HR = 2.223, 95% CI, 1.031–4.797; *p* = 0.042), DM (HR = 2.221, 95% CI, 1.029–4.791; *p* = 0.042), obesity (HR = 2.519, 95% CI, 1.095–5.795; *p* = 0.030), and old age (>65 years old) (HR = 2.302, 95% CI, 1.044–5.075; *p* = 0.039). After adjustment of all variables, which is statistically significant in the univariate analysis, the multivariate predictors of HFE were still rest PFR (HR = 2.766, 95% CI, 1.244–6.150; *p* = 0.013), DM (HR = 2.201, 95% CI, 1.014–4.773; *p* = 0.013), obesity (HR = 3.332, 95% CI, 1.395–7.960; *p* = 0.007), and old age (HR = 2.631, 95% CI, 1.174–5.899; *p* = 0.019).

**Table 4:** Independent predictors of HFE.

**Predictors**	**Independent HR**	***p*-Value**	**Adjusted HR**	***p*-Value**
Rest PFR	2.223 (1.031–4.797)	0.042	2.766 (1.244–6.150)	0.013
Stress PFR	1.732 (0.803–3.738)	0.162		
Ischemia-related DD	1.208 (0.455–3.204)	0.705		
DM	2.221 (1.029–4.791)	0.042	2.201 (1.014–4.773)	0.013
Obesity	2.519 (1.095–5.795)	0.030	3.332 (1.395–7.960)	0.007
Old age	2.302 (1.044–5.075)	0.039	2.631 (1.174–5.899)	0.019

*PFR, peak filling rate; DD, diastolic dysfunction; DM, diabetes mellitus*.

## Discussion

The objective of the present study was to determine the prognostic value of DD derived from D-SPECT in CAD patients with normal ejection fraction. Our major findings were (1) CAD patients with normal ejection fraction and DD derived from D-SPECT were associated with worse prognosis, and (2) the multivariable predictors of HFE in CAD patients with normal ejection fraction were obesity, DM, and rest DD.

Echocardiography has by far played a central role in the evaluation of DD, and there have been definitive diagnostic criteria for DD recommended from the ASE guideline (10). DD is present if more than half of the available recommended parameters meet their cut-off values including septal e′ <7 cm/s, lateral e′ <10 cm/s, average E/e′ ratio >14, left atrial volume index >34 ml/m^2^, and peak tricuspid regurgitation velocity >2.8 m/s. Previous studies demonstrated the feasibility to quantitate the diastolic function parameters with SPECT, and the thresholds for abnormal PFR and abnormal TTPF were 1.7 EDV/s and 208 ms, respectively (18, 23). Compared with conventional SPECT, D-SPECT with dedicated cardiac CZT cameras has more favorable sensitivity, specificity, and accuracy with a low-dose and ultra-fast protocol (13–15). However, there is no optimal cut-off value recommended from guideline for PFR to diagnose DD with D-SPECT. A previous study stipulated that the presence of abnormal diastolic function assessed by CZT-SPECT existed when PFR was <2.0 EDV/s (24). A recent study demonstrated that PFR and TTPF derived from CZT-SPECT showed a good correlation with the diastolic function parameters obtained by echocardiography (11). Accordingly, PFR <2.1 EDV/s was selected as the definition of an abnormal result in the present study. Despite the lack of an optimal threshold that reflects abnormal PFR, the primary concept is that a low PFR is indicative of abnormal diastolic function, which represents risk of future adverse clinical outcomes. Since there is a lack of confirmation of cut-off value for DD, further studies should be done to confirm the threshold of PFR.

It is validated that myocardial ischemia is associated with DD derived from echocardiography (10, 25). One of the clinical manifestations of myocardial ischemia is abnormal relaxation and DD (26, 27). Previous studies showed that in the community population, DD defined by echocardiography is common and is associated with significant increase in all-cause mortality (1, 28). Moderate to severe DD was present in 10% of stable CAD patients with normal ejection fraction, and no history of HF is predictive of subsequent hospitalization for HF and incidence of cardiovascular and all-cause death (8, 9). A recent study demonstrated that positive diastolic stress test assessed by echocardiography predicted more HFE than negative diastolic stress test (29). In addition, the severity and extent of non-obstructive and also obstructive CAD is associated with measures of worsening diastolic function (7). Furthermore, the survival of patients with HF with preserved ejection fraction was comparable with that of patients with reduced ejection fraction (30, 31). Therefore, early identification of DD might be of significant value for risk stratification of CAD patients with normal ejection fraction. Despite the poor prognosis of DD assessed by echocardiography is validated, the prognosis of DD derived by SPECT is lacking. This study was designed to evaluate the prognostic value of diastolic function derived from D-SPECT in CAD patients with normal systolic function. The present study showed that CAD patients with DD and normal ejection fraction were associated with worse prognosis. Despite the validated association between DD and myocardial ischemia, our results showed that there were no significant differences in ischemic-related indicators from MPI between three groups. One of the surprising results was that the prognosis of patients with abnormal stress PFR and normal rest one was better than those with abnormal rest PFR. Patients with rest DD predicted more HFE. The principal reason for this phenomenon might be that in the present study, stress testing was performed with pharmacological stress, and as a result, the prognosis might be different with those undergoing treadmill exercise stress. To the best of our knowledge, the present study is the first study to evaluate the prognostic value of diastolic parameters assessed by D-SPECT. Further large-scale studies and multicenter study design are required to confirm the results we obtained.

Several limitations should be considered in the present study. One of the primary limitations is that this study was a retrospective and observational analysis of diastolic function assessment with a D-SPECT camera, which accompanies an inherent bias due to the type of study design. Second, the final results might be interfered with percutaneous coronary intervention (PCI) and the severity of coronary obstruction, and further studies with specific classification for CAD patients should be performed to avoid this problem. In the present study, we did not confirm the correlations between echocardiography indices of diastolic function and D-SPECT derived diastolic indices, which is necessary to define a PFR threshold for DD. Furthermore, we could not provide any information related to DSPECT and CAG comparisons among our patients. Compared with the previous studies, the follow-up period of the present study was relatively short, which could have led to under-reporting of HFE. This study is single-centered, and the results might not be applicable in other research and camera designs, i.e., both CZT and conventional imaging. Consequently, multicenter randomized control trials and studies with more rational design are required to verify the results of the present study.

## Conclusion

DD derived from D-SPECT in CAD patients with normal ejection fraction is predictive of HFE and worse prognosis.

## Data Availability Statement

The data analyzed in this study is subject to the following licenses/restrictions: Hospital regulations. Requests to access these datasets should be directed to chewenliang@tongji.edu.cn.

## Ethics Statement

The studies involving human participants were reviewed and approved by Shanghai Tenth People's Hospital, Tongji University, Shanghai, China. The patients/participants provided their written informed consent to participate in this study. Written informed consent was obtained from the individual(s) for the publication of any potentially identifiable images or data included in this article.

## Author Contributions

BX, FA, WC, HC, and FY: designed the study. LL, BX GY, A-QM, and SX: collected the data. BX, XL, RF, CF, TS, and WZ: were involved in data cleaning, follow-up, and verification. FA and WC: analyzed the data. BX, FA, YX, and WC: drafted the manuscript and revised it critically for important intellectual content. WC: approved the final version of the manuscript. All authors contributed to manuscript revision, read, and approved the submitted version.

## Conflict of Interest

The authors declare that the research was conducted in the absence of any commercial or financial relationships that could be construed as a potential conflict of interest.
