# Supplementary figures and images for: Prognostic Value of Diastolic Dysfunction Derived From D-SPECT in Coronary Artery Disease Patients With Normal Ejection Fraction
Source: Front Cardiovasc Med. 2021 Jul 15;8:700027. doi: 10.3389/fcvm.2021.700027 (PMC8319539; doi:10.3389/fcvm.2021.700027)

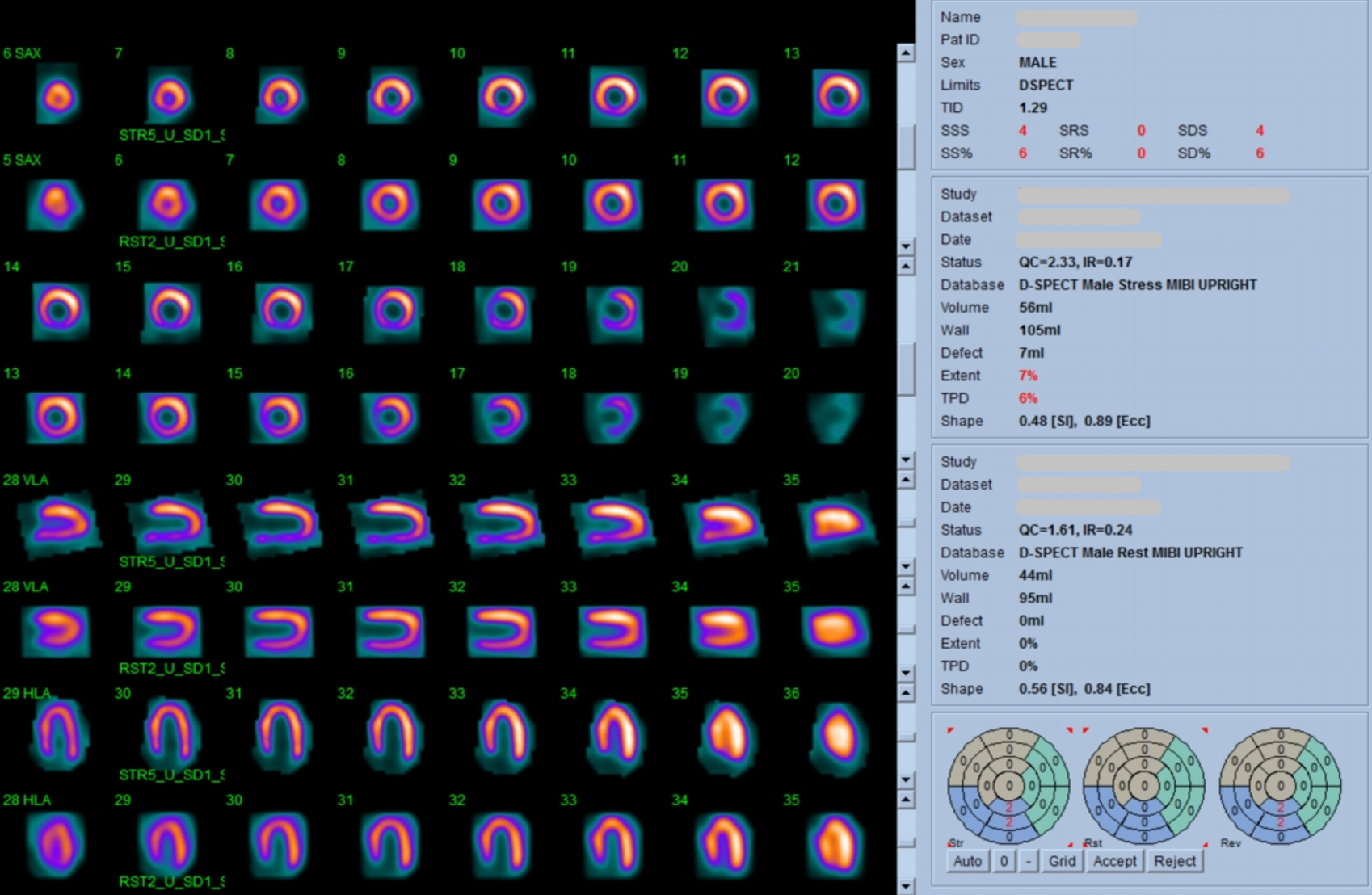

Supplement: Supplementary file 1 [file Image_1.PNG]

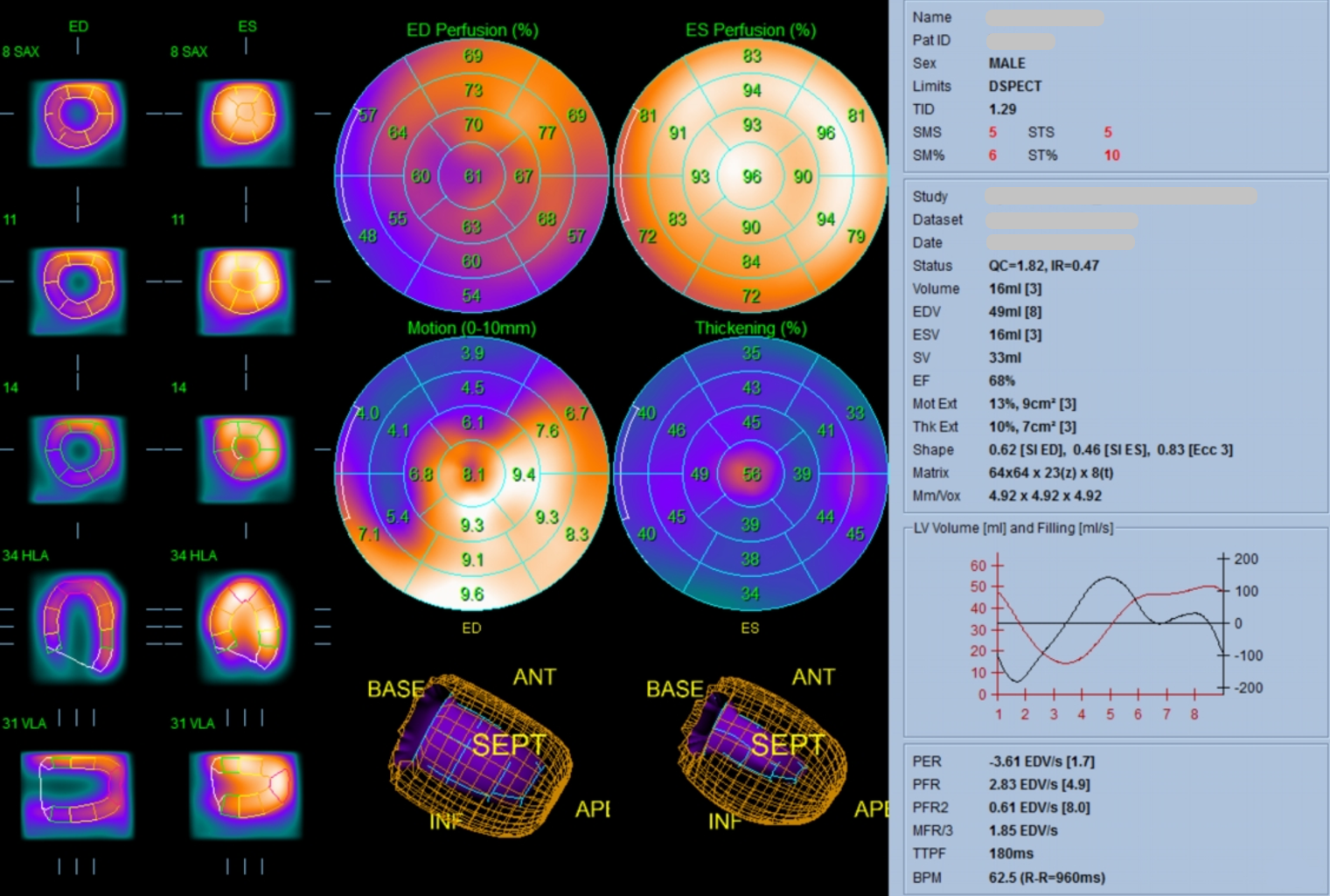

Supplement: Supplementary file 2 [file Image_2.PNG]
